# Biological Control Services from Parasitic Hymenoptera in Urban Agriculture

**DOI:** 10.3390/insects13050467

**Published:** 2022-05-17

**Authors:** Joshua Earl Arnold

**Affiliations:** 1 Department of Environmental Science, Policy and Management (ESPM), University of California—Berkeley, Berkeley, CA 94720, USA; joshua-arnold@warren-wilson.edu; 2 Department of Environmental Studies, Warren Wilson College, Asheville, NC 28815, USA

**Keywords:** agroecological pest management, conservation biological control, ecosystem services, parasitic Hymenoptera, urban agriculture

## Abstract

**Simple Summary:**

Our findings support the enemies hypothesis in urban agroecosystems. Local factors, including increased mulch coverage, crop richness, and percent of non-crop areas, are predictors of increased PH abundance and aphid parasitism rates. Our findings support and strengthen previous findings in UA research. Urban farmers should be encouraged to diversify urban agroecosystem spatial composition and implement APM practices to reduce pest impacts.

**Abstract:**

Urban agriculture is practiced in spatially fragmented landscapes with unique characteristics that can impact species occurrence in time and space. As a result, biological control services, an ecosystem service from naturally occurring arthropod natural enemies, can be negatively impacted. Many urban farms forgo pesticides and utilize agroecological pest-management strategies that rely on natural enemies to help regulate pest populations. Understanding how these enemies are affected by landscape composition and on-farm management practices is critical to understanding agroecological pest management in UA and furthering our understanding of landscape-mediated population dynamics. Over two growing seasons, we sampled brassica crops in urban agriculture sites occurring on a spectrum of surrounding landscape imperviousness, spatial composition, size, and management practices to better understand parasitic Hymenoptera abundance, richness, and parasitism rates on the common cabbage aphid (*Brevicoryne brassicae*). We found that on-farm agroecological pest-management practices such as mulch coverage, floral richness, and overall crop-plant richness impacted parasitic Hymenoptera abundance. Larger proportions of on-farm noncrop area increased parasitoid abundance on urban farms. Aphid parasitism increased in relation to on-farm management practices, including increased crop-plant richness. These findings add to a growing understanding of urban agroecosystem function and support the enemies hypothesis in urban agroecosystems.

## 1. Introduction

Crop pests in urban landscapes can be challenging to control, and they can have a disproportionate impact on the smaller crop sizes common to urban farms. Herbivorous insect populations in urban areas can persist for more extended periods, have increased fecundity, and can even be larger [1,2,3,4,5,6]. Undermanaged or neglected urban landscapes can exacerbate pest issues. Irregular irrigation, application of fertilizers or pesticides, and higher levels of air pollution can induce plant stress or vigor, creating favorable conditions and refuges for herbivorous pests that can emigrate to urban farms and gardens [7,8]. Urban agriculture (UA) is often practiced without pesticides for health and environmental reasons despite these challenges. Instead, farmers find themselves relying on time and labor-intensive cultural and mechanical practices for pest management. Consequently, urban farmers have shown great interest in agroecological pest management (APM), a proactive ecosystem-services-based approach that aims to reduce pest abundance and crop damage by increasing natural enemy populations through agroecological practices [9,10]. These practices increase the amount of shelter, nectar, and pollen resources on urban farms, increasing natural enemy populations, resulting in increased biological control services [11].

In rural agroecosystems, APM practices, landscape effects, and conservation biological control have been widely studied [12,13]. Meta-analyses have found that on-farm management practices such as intercropping, crop rotations, and increased structural diversity increase natural enemies’ abundance, diversity, and ability to regulate pest populations [13]. Increased landscape diversity surrounding rural agroecosystems has been shown to mediate arthropod diversity and abundance, with natural enemies showing a positive response to increased landscape complexity [12]. The enemies hypothesis states that increased structural complexity should increase natural enemy abundance, diversity, and associated ecosystem services [14,15,16,17]. This hypothesis has been investigated and questioned in agroecosystem management, with varying results at different spatial and temporal scales, most often in rural contexts. Ostensibly, diversification effects observed in rural agroecosystems should be observed in their urban counterparts. However, the effects of diversification on biological control services and APM in urban agroecosystems regarding this hypothesis are still being explored, especially in how natural enemies are affected by landscape factors such as fragmentation and isolation, common in urban landscapes [18]. 

The extent of fragmentation effects on organisms in urban environments, and related ES, has been a persistent question, especially in urban agroecosystem management [19]. Roads, parking lots, and buildings increase impervious surfaces, fragmenting and reducing greenspace connectivity and impacting the quality and area of suitable habitat [20,21]. The reduction in available and appropriate habitats for urban flora and fauna decreases metapopulation connectivity and drives a decline in urban species diversity, selecting for more disturbance-tolerant species and increasing the chance of localized extinctions [20,22,23,24,25]. Existing literature on the effects of urbanization on species occurrence, abundance, and diversity often relies on urban–rural gradient studies [19,26,27]. These studies generally find that increased urbanization decreases the diversity of organisms [23,28]. Confirming these findings are an abundance of patch-matrix literature suggesting that the quality of the habitat patch itself, its size, and the composition of the matrix surrounding it are determining factors for species occurrence in fragmented landscapes [20,25,29,30]. Specific to UA, higher imperviousness surrounding urban farms has been related to decreased parasitoid abundance and richness [31,32], decreased predator abundance and richness [33,34,35], and even decreased predation on sentinel prey [36]. 

While landscape effects in UA have also been shown to increase abundance [34,37,38] and diversity [31,34,39] of natural enemies, the composition of the overall matrix in urban areas is often outside of the scope of management of urban farmers. However, patch quality is easily manipulated through management practices. Abundance of perennials, height of herbaceous cover, and amount of seminatural or noncrop area on urban farms have been measured in UA and have been shown to positively impact a wide diversity of natural enemies [18]. Area of ground cover, especially mulch cover, has been correlated with increased natural enemy abundance [31,32,37] and richness [31,39]. Increased proportions of complex ground covers have been associated with increased rates of prey removal in sentinel prey trials in urban gardens [36]. Increased floral abundance and diversity has been shown to increase natural enemy abundance [32,35,40,41] and richness [26,40,41]. Moreover, spatial configuration of working landscapes has become an increasingly important aspect of species occurrence and related biological control services [24,42]. Understanding how landscape and management may affect natural enemy abundance and diversity of natural enemies is an important aspect of effective APM in UA [30,43,44,45].

This research continues to build on previous findings from studies of local and landscape effects on biological control services in UA by focusing on parasitic Hymenoptera (PH) in urban agroecosystems. PH are important in provisioning biological control because they utilize arthropod hosts during their juvenile life stages, leading to the termination of hosts. Previous studies focused on UA in the context of local and landscape effects on PH have found both increased and decreased abundance with higher rates of imperviousness between 200–500 m [26,31,37,40], and with larger gardens [32,41,46]. APM practices, including floral provisioning, have been shown to increase both abundance and diversity of PH [40,41]; increased mulch coverage has been shown to increase PH abundance [31,32]; and structural diversity increases PH richness [31,34]. Due to the mixed results of pervious findings, especially in the context of potential beneficial affects to APM practices, further research is necessary. 

This research focuses on PH-mediated biological control services in brassica cropping systems in UA. Specifically, we focus on intraurban effects (landscape composition, on-farm spatial composition, and management practices) on the abundance of different taxa of PH and the parasitism of aphids. We attempt to clarify previous findings by focusing solely on brassica cropping systems ubiquitous across urban farming systems in the San Francisco Bay Area, USA. We hypothesize that urban farmers can increase on-farm biological control services by controlling for patch quality through agroecological pest-management practices, further supporting evidence for the enemies hypothesis in fragmented landscapes. To test this hypothesis, we investigate whether APM practices (mulch coverage, floral richness, and increased crop richness) significantly affect the abundance of PH in UA. Secondly, we question if surrounding imperviousness and on-farm spatial composition influence biological control services. Lastly, we question whether APM practices impact PH abundance and biological control services to a greater extent than landscape factors. 

## 2. Methods

### 2.1. Field Sites and Sampling

To better understand PH richness and abundance in urban farms and associated biological control services, we conducted an in-situ survey at urban community farms in the East Bay of the San Francisco Bay Area, USA. Eleven farms participated in 2018 and ten farms in 2019. Farms were asked to participate in research based on two factors: (1) farm size, to ensure a comparative sample of small, medium, and large farms; and (2) high or low levels of surrounding impervious surface per the National Landscape Cover Database (NLCD) (see Appendix A). Landscape factors and APM practices of farms were measured. APM practices included area of non-crop usage (includes all non-crop vegetation), area of production, crop-plant abundance (brassica), crop richness, floral richness, and percent of farm surface with complex ground covers including mulch and leaf litter. Landscape factors included percent of impervious surface at 200-, 500-, and 1000-m radii. Sampling iterations occurred from May to mid-October each year. 

On-farm non-crop area was defined as a not actively managed area of the farm occupied by non-crop flora. Farm size in m^2^ was calculated through Google Earth Pro and ground-proofed during on-farm spatial measurements. Brassica abundance was determined by counting all brassicas on the farm when sampling occurred. Crop-plant richness was determined by eight-meter transects measured perpendicular to garden beds three times during the growing season. Different cultivars of the same species (e.g., kale and broccoli) were counted separately when measuring crop richness. Floral richness was surveyed three times per growing season (early, mid, and late) by completing a comprehensive count of each flowering plant at each survey site. Randomized 4 m^2^ quadrats were used to estimate percent of and type of cover (woody mulches or leaf litter). Ground cover quadrats were measured across crop and non-crop areas. Percentages of surrounding impervious surfaces (e.g., pavement, buildings, or other structures) for each farm were measured using the NLCD at 8 m resolution (see Appendix A).

Collection of PH was accomplished by using an insect vacuum on *Brassica oleracea* cultivars, including broccoli, kale, collards, and tree collards. Each sampled plant was randomly selected and was only sampled if it was standing free of other herbaceous cover and flowering plants. A total of nine plants of each cultivar present were sampled per visit. Vacuum sampling occurred monthly from May to October. Vacuuming of each plant lasted for five seconds. For this work, we assume that sampled wasps were performing foraging or host-seeking behaviors on selected plants [47]. Each sample was frozen until processed by extracting all PH and identifying them to the lowest taxonomic level possible per previous literature [26,31,34]. PH identification was accomplished using Hymenoptera of the World [48]. Chalcidoidea were identified with the Annotated keys to the Genera of Nearctic Chalcidoidea (Hymenoptera) [49], and Braconidae using the Manual of the New World Genera of the Family Braconidae [50]. Collected specimens that were damaged were identified to the closest identifiable morphospecies. *Cabbage aphids*, *Brevicoryne brassicae*, were visually identified, and abundance was assessed by conducting a total count on three random leaves on nine brassicas per cultivar, including counts of apterous, alate, and parasitized aphids. Aphid abundance counts were performed monthly from May to October on non-vacuum-sampling days to reduce PH disturbance. Parasitism rates were calculated as number of parasitized aphids divided by number of total aphids on each leaf.

### 2.2. Data Analysis

Generalized linear mixed models (GLMM) were constructed using the MASS R package [51] to explore whether APM practices or landscape factors affected PH abundance on common brassicas. Each response variable: All PH, PH superfamily, family, and subfamily abundance, overall site PH diversity, and rates of aphid parasitism were modeled with both local and landscape factors. Local factors include the percent of mulch ground cover, floral and crop richness, production, and non-crop area. Landscape factors include percentage of impervious surface at 200, 500, and 1000 m radii, and farm size. Seasonal factors included both year and season and were assessed as categorical variables: early season (May–June), midseason (July–August), and late season (September–October). The fitdistrplus package in R was used to find appropriate distributions for modeling [52]. A negative binomial or Poisson distribution with a log-link function was selected as appropriate given the zero inflation of the count data. Models were fitted with the glmer.nb or glmer function in R package MASS [51]. Preliminary models with all measured local and landscape factors were constructed for each response variable. Explanatory variables of low importance for all response variables were excluded from subsequent models. Final models (See Appendix A) were assessed for fit using the Akaike Information Criterion (AIC) and diagnosed for over- or underdispersion by comparing observed residuals with expected residuals using the DHARMa package in R. Poorly fitted models were excluded from the results [53]. Partial regression plots (predictor effect plots) for final models were developed using the “effects” package in R and are reported in Results [54]. The slope of the line in these plots represents the association between a single explanatory variable and a response variable accounting for the effects of each other variable within the fitted model. 

## 3. Results

### 3.1. Parasitic Hymenoptera Sampling

Nine hundred and thirty-eight total vacuum samples yielded 2048 individual PH in the period over 2018–2019. We identified six superfamilies of PH: Ceraphronoidea, Chalcidoidea, Cynipoidea, Ichneumonoidea, Platygastroidea, and Proctotrupoidea, twenty-seven families and fifty-one subfamilies. Our most commonly sampled taxa included the family Braconidae (*n* = 852), and the superfamily Chalcidoidea (*n* = 582), both widely used historically in biological control efforts. The Braconidae included two main subfamilies, Aphidiinae (*n* = 813) and Opiinae (*n* = 39). Sampled families of Chalcidoidea included Pteromalidae (*n* = 224), Aphelinidae (*n* = 136), Eulophidae (*n* = 133), Eucharitidae (*n* = 27), and Encyrtidae (*n* = 19). Four hundred and thirty-three Cynipoidea were collected, including the family Figitidae (*n* = 90), cynipoid subfamily Charipinae (*n* = 59), and the family Eucoilidae (*n* = 47). Three superfamilies including two families and one subfamily were collected in sufficient numbers to be included in the analysis (Table 1). 

### 3.2. Influence of APM Practices and Local Factors on Parasitic Hymenoptera Abundance and Aphid Parasitism

Final GLMM models (See Appendix A) showed significant effects of local and seasonal variables on the sum abundance of several PH taxa at the superfamily, family, and subfamily levels and the parasitism of aphids. No landscape variables had any effect on PH abundance or rates of aphid parasitism. 

### 3.3. All Parasitic Hymenoptera

**Figure 1:**
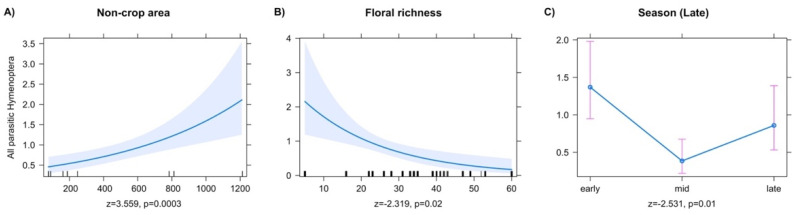
Predictor effect plots for individual explanatory variables on the abundance of all Parasitic Hymenoptera.

Models for the abundance of all collected parasitic Hymenoptera showed significant effects of season and local APM factors. The abundance of all PH collected increased with larger noncrop areas on the farm (Figure 1A). All PH abundance decreased with increased floral richness (Figure 1B). Despite an increase in collected PH in 2019 (2018, *n* = 872 and 2019, *n* = 1007), models that included season as an explanatory variable (early, mid, and late) and year (2018 or 2019) showed a significant overall decrease in PH abundance in late season (Figure 1C, z = −2.531, *p* = 0.011).

### 3.4. Superfamily Chalcidoidea and Family Aphelinidae 

**Figure 2:**
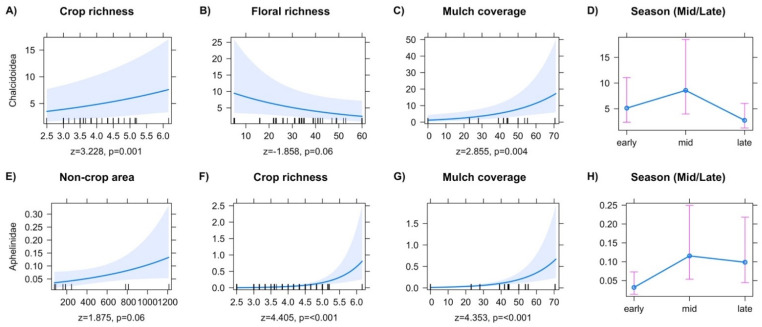
Predictor effect plots for individual explanatory variables on the abundance of superfamily Chalcidoidea (**A**–**D**), and family Aphelinidae (**E**–**H**).

The Chalcidoidea and Aphelinidae showed significant responses in abundance to local explanatory variables. Final models for Chalcidoidea predicted both positive and negative responses in abundance to local factors, including increased abundance with increased crop richness (Figure 2A), and reduced abundance with increased on-farm floral richness (Figure 2B). Increased mulch coverage was associated with increased chalcidoid abundance (Figure 2C). Models for the family Aphelinidae showed significant effects from local variables, including increased abundance with increasing non-crop area (Figure 2E), crop richness (Figure 2F), and mulch coverage (Figure 2G). Chalcidoidea had a midseason increase (Figure 2D, z = 4.215, *p* ≤ 0.001), and late-season decrease in abundance (Figure 2D, z = −3.947, *p* ≤ 0.001). Aphelinidae abundance increased in both the mid (Figure 2H, z = 2.248, *p* ≤ 0.024), and late season (Figure 2H, z = 1.904, *p* = 0.056).

### 3.5. Superfamily Cynipoidea

**Figure 3:**
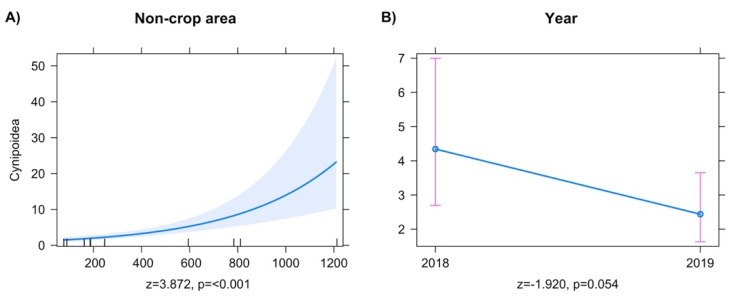
Predictor effect plots for individual explanatory variables on the abundance of superfamily Cynipoidea.

Final models for Cynipoidea showed an increase in abundance with a greater noncrop area (Figure 3A), and an overall reduction in abundance between 2018 and 2019 sampling periods (Figure 3B).

### 3.6. Family Braconidae and Subfamily Aphidiinae

**Figure 4:**
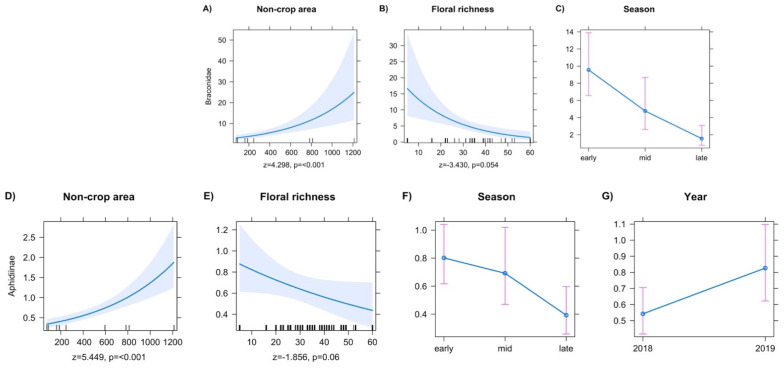
Predictor effect plots for individual explanatory variables on the abundance of family Braconidae (**A**–**C**), and subfamily Aphidiinae (**D**–**G**), and family Aphelinidae (Figure 2E–H).

Final models for Braconidae showed a positive response in abundance to increased non-crop area (Figure 4A). Floral richness reduced braconid abundance (Figure 4B). Models for aphidiine abundance included local and temporal explanatory variables in the final model. Increases in the local factors’ non-crop area increased aphidiine abundance (Figure 4D). Floral richness reduced aphidiine abundance (Figure 4E). Aphidiine wasps had a lower abundance in the late season over both sampling years (Figure 4F, z = −2.841, *p* = 0.004), but generally had a greater abundance in samples during 2019 (Figure 4G, z = 2.13, *p* = 0.033). Across both sampling years, braconid abundance was reduced in both the mid (Figure 4C, z = −1.971, *p* = 0.048), and late season (Figure 4C, z = −4.615, *p* ≤ 0.001).

#### Aphid Parasitism

**Figure 5:**
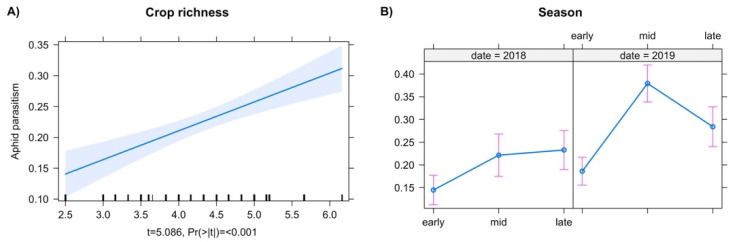
Predictor effect plots for individual explanatory variables. (**A**) Crop richness, (**B**) season and year on rates of parasitism.

Rates of aphid parasitism increased with crop richness (Figure 5A). In addition, parasitism rates varied greatly in 2019, with the highest levels measured in mid- (Figure 5B, t = 7.371, *p* = 0.0001) and late-season 2019 (Figure 5B, t = 4.897, *p* = 0.0001).

## 4. Discussion

To test the local and landscape effects on the enemies hypothesis vis-a-vis APM on populations of PH in urban agroecosystems, we collected data from twelve urban farms in the San Francisco Bay Area over a period of two growing seasons. Participating farms were selected to represent a continuum of size, spatial composition, and surrounding imperviousness. Non-crop area was a significant predictor for all PH, cynipoid, and braconid wasps. Effects of APM practices were varied, but increased crop richness and mulch coverage were associated with increased abundance of all Chalcidoidea, including the Aphelinidae. Increases in crop richness also showed an increase in parasitism rates of aphids on brassica crop plants. Unexpectedly, floral richness showed a negative relationship to the abundance of all PH, as well as chalcids, and all Braconidae. All PH showed a significant decline in abundance during the late season of 2019. All measures of impervious surface surrounding urban farms had no effect on PH abundance or aphid parasitism on the urban farms. Landscape effects to arthropod-mediated ES continue to have mixed results and this research supports previous findings in urban agriculture, which show both negative and positive effects to natural enemy abundance and diversity [55]. 

### 4.1. On-Farm Spatial Composition

Non-crop areas identified in this research are difficult to identify explicitly as either managed or unmanaged, and existed on a spectrum that was often difficult to quantify in interviews or through survey work. However, these areas had most frequently been improved with flowering perennials or annuals and medicinal or “native” flora, and farmers typically stated the purpose as providing a resource for wildlife or beneficial insects. Previous research supports farmer efforts. Structural diversity has been found to elicit positive responses with regard to diversity and abundance of predators and PH in previous UA studies [31,34,36,56]. These areas may provide critical overwintering habitat in annual cropping systems, additional hosts or prey, shelter, and floral nectar resources for nectarivorous insects [11,14]. Our findings suggest that these non-crop areas have the potential to influence agroecosystem function in UA, and may be an important part of APM practices, even in highly fragmented landscapes. Moreover, floral richness had little effect on PH abundance, or parasitism of aphids, signaling that increases in PH abundance were not due to floral nectar within these non-crop areas. Another mechanism that may be of importance are the spatial composition (or configuration) of the agroecosystem. Our research did not take into account the overall distribution of the non-crop area within the farm, which may have failed to account for spatial heterogeneity that has been found to elicit positive and negative biological control responses in agroecosystems [42]. Future research on urban farms should account not only for the proportion of non-crop areas, but also spatial heterogeneity to further explore these effects. 

### 4.2. APM Practices

Overall, APM practices, such as increased mulch coverage and crop-plant richness were important predictors of PH abundance, and increased aphid parasitism rates. The connection between mulch, complex groundcovers, and increased abundance and diversity of parasitic wasps has been previously observed in urban agroecosystems [31,32], a variety of natural habitats, and rural agroecosystems [57]. It is unlikely that mulch would provide a direct resource for PH, but PH may benefit from mulch as a potential overwintering habitat or it may provide habitat for potential hosts. Many of the collected PH were parasitoids of dipteran larvae; these larvae are herbivorous but complete part of their life cycle in soils. I suggest that the overall biodiversity of urban farms with increased mulch coverage may create a bottom-up trophic cascade that increases overall soil arthropod diversity benefiting PH populations. 

Floral richness had a negative effect on PH abundance in all models. Floral richness was chosen as an explanatory variable, as it has previously been found to increase PH abundance in UA [41]. The vast majority of PH are nectarivorous, and this additional nectar resource has been suggested frequently as a strategy for increasing populations, potentially leading to increased parasitism [11,14,57,58]. However, conflicting data raise questions about this on-farm manipulation and whether PH seek hosts in the same area they feed, or they disperse to increase fecundity [59]. A large proportion of our overall sample of PH were cynipoids, potentially from the genus *Alloxysta*, known hyperparasitoids of both dominant primary aphid parasitoids in our sample, Aphidiinae, and Aphelinidae [60]. These reductions in primary aphid parasitoid populations may be due to direct or indirect negative effects from this hyperparasitoid that also feeds on floral nectar [59,61,62]. In urban agroecosystems, floral provisioning as a habitat manipulation may be complicated by the inherent fragmentation and quality of the urban matrix. For floral resources to be an effective APM practice, this resource must be limited. Potential concentrations of alternate off-farm floral resources may complicate this affect. 

Crop-plant richness positively affected the abundance of all Chalcidoidea and the subfamily Aphelinidae. Crop richness was also a predictor of greater parasitism rates of cabbage aphids on sampled brassica. Similar findings in rural and urban agroecosystems, including increased PH abundance and biological control services in relation to increased crop diversity have been previously documented [13,31,35,38,56,63]. Given that intercropping is commonly practiced in UA, these results validate the efficacy of the practice, and offer an opportunity to investigate the extent of the effect in future research efforts.

While this research expanded upon previous findings and can be of utility for urban agroecosystem management, questions remain. Notably, the effects of hyperparasitism on biological control in UA. Our third most collected taxon was Cynipoidea, many of which are often hyperparasitoids of aphid-parasitizing wasps [64]. Given that these cynipoids were collected from plant foliage in close proximity to many primary aphid parasitoids, there is some anecdotal evidence that these cynipoids were engaging in host-seeking behavior. If some of the measured on-farm management practices, such as increased noncrop areas, also increase abundance of Cynipoidea, this could result in decreased biological control services. In this case, floral provisioning may potentially be acting as an ecosystem disservice [59,61,65,66]. Unfortunately, we were unable to collect parasitized aphids and rear any hyperparasitoids during this research, but these findings suggest that hyperparasitism in fragmented UA landscapes may be a mechanism affecting APM strategies in UA. 

### 4.3. Seasonal Factors

Seasonal effects on PH abundance were mixed, but many effects were measured in the second year of our sampling. Of note, in 2019, we had fewer sampling events as one farm was unable to participate in our study, but more PH were collected in that year despite the smaller sampling pool. Rates of aphid parasitism were significantly decreased between the mid and late season in 2019. It is unknown what drove these effects, but it is notable that such a significant difference could occur between sampling seasons. Future research efforts should consider seasonal differences and weather when drawing conclusions about on-farm or landscape factors to PH abundance or diversity or associated biological control services.

## Figures and Tables

**Table 1:** Overview of PH analyzed.

Parasitic Hymenoptera (Data Analysis)			
Superfamily	Family	Subfamily	*n* =
Chalcidoidea (All) *	*-*	*-*	582 ^A^
Chalcidoidea	Aphelinidae *	Unk.	136
Chalcidoidea	Eulophidae	Unk.	133
Chalcidoidea	Eulophidae	Entedoninae	23
Chalcidoidea	Pteromalidae	Unk.	224
Cynipoidea (All) *	*-*	*-*	464 ^A^
Cynipoidea	Figitidae	Charipinae	59
Cynipoidea	Figitidae	Unk.	31
Cynipoidea	Eucoilidae	Unk.	47
Ichneumonoidea	Braconidae *	*-*	852 ^A^
Ichneumonoidea	Braconidae	Aphidiinae *	813
Ichneumonoidea	Braconidae	Opiinae	39

* = Included in final GLMM models. ^A^ = Total number of specimens per superfamily.

## Data Availability

The data presented in this study are available on request from the corresponding author.

## Appendix A

Farm characteristics table.

**Table A1:** Farm spatial characteristics and location.

Site Coordinates (Plus Code)	Farm Size (m^2^)	Production Area (m^2^)	Noncrop Area (m^2^)	Impervious Surface % (200 m Radii)	Impervious Surface% (500 m Radii)	Impervious Surface% (1 km Radii)
VMJX + P8 Berkeley, California	8903	1028	784	69	67	67
VPCR + QH Berkeley, California	5712	2300	0	75	67	62
RPC7 + VJ Oakland, California	5308	381	1213	84	78	80
WMWM + 4F Richmond, California	4477	966	595	56	49	41
QR5P + PQ Oakland, California	3892	1998	162	62	61	57
VP87 + 2J Berkeley, California	2348	760	188	66	60	61
VPH6 + FF Berkeley, California	2299	1007	277	55	57	58
XM23 + R6 Richmond, California	1428	206	77	34	57	64
QR27 + MX Oakland, California	968	111	247	64	71	75
QPVQ + V8 Oakland, California	932	140	52	52	64	74
VP39 + 46 Berkeley, California	799	178	247	63	62	64

**Table A2:** Final GLMM Models.

Final Models	AIC
All parasitic Hymenoptera ~ Date + Season + Average mulch coverage + Crop richness + Floral richness + Noncrop area + Site *	1425
All Chalcidoidea ~ Date + Season + Average mulch coverage + Crop richness + Floral richness + Site *	529
Chalcidoidea Aphelinidae ~ Date + Season + Average mulch coverage + Crop richness + Total size + Production size + Site *	331
All Cynipoidea ~ Date + Season + Crop richness + Noncrop area + Site *	407
Ichneumonoidea Braconidae ~ Date + Season + Average mulch coverage + Crop richness + Floral richness + Site *	332
Ichneumonoidea Braconidae Aphidiinae ~ Date + Season + Floral richness + Noncrop area + Site *	1768
Rate of parasitism ~ Date * season + Crop richness ^A^	−143

* = Site as random effect to control for pseudoreplication. ^A^ = Modeled as GLM as the variable “site” had no effect.

**Table A3:** GLMM Results.

Response Variable	Explanatory Variable	Est.	Std. Err	z-Value	Pr (>|z|)
All parasitic Hymenoptera	Area of noncrop	0.0013	0.0004	3.559	0.000372
-	Floral richness	−0.031483	0.0136	−2.319	0.020402
-	Season (Late)	−0.828814	0.3274	−2.531	0.011363
Chalcidoidea	Crop richness	0.2099	0.065	3.228	0.00125
-	Floral richness	−0.0247	0.0133	−1.858	0.06311
-	Mulch coverage	0.0377	0.0132	2.855	0.0043
-	Season (Mid)	0.5145	0.1221	4.215	0.000025
-	Season (Late)	−0.62076	0.1573	−3.947	0.000079
Aphelinidae	Area of noncrop	0.0012	0.0006	1.875	0.0608
-	Crop richness	1.4113	0.3204	4.405	0.000011
-	Mulch coverage	0.0589	0.0135	4.353	0.000013
-	Season (Mid)	1.29	0.5738	2.248	0.0246
-	Season (Late)	1.1325	0.5947	1.904	0.0569
Cynipoidea	Area of noncrop	0.0023	0.0006	3.872	0.000108
-	Year (2019)	−0.650749	0.3389	−1.92	0.05481
Braconidae	Area of noncrop	0.0018	0.0004	4.298	0.000017
-	Floral richness	−0.044612	0.013	−3.43	0.000603
-	Season (Mid)	−0.695516	0.353	−1.971	0.048778
-	Season (Late)	−1.820861	0.3945	−4.615	0.000004
Aphidiinae	Area of noncrop	0.0015	0.0003	5.449	5.08 × 10^−8^
-	Floral richness	−0.012649	0.0068	−1.856	0.06345
-	Season (Late)	−0.714486	0.2515	−2.841	0.0045
-	Year (2019)	0.4209	0.1976	2.13	0.0332
		Est.	Std. err	t-Value	Pr (>|t|)
Aphid parasitism ^A^	Crop richness	0.0468	0.0092	5.086	4.59 × 10^−7^
-	Date (2019):season (Mid)	0.1419	0.0193	7.371	4.34 × 10^−13^
-	Date (2019):season (Late)	0.0942	0.0192	4.897	4.34 × 10^−13^

^A^ = When modeled in GLMM, the random effect (Site) was not present. Therefore, modeling for rates of parasitism were completed with GLM.
